# Visual LAMP method for the detection of *Vibrio vulnificus* in aquatic products and environmental water

**DOI:** 10.1186/s12866-022-02656-1

**Published:** 2022-10-21

**Authors:** Zhuo Tian, Lili Yang, Xin Qi, Qiuyue Zheng, Dejing Shang, Jijuan Cao

**Affiliations:** 1 Liaoning Normal University, Dalian, 116023 China; 2 Key Laboratory of Biotechnology and Bioresources Utilization of Ministry of Education, Dalian Minzu University, Dalian, 116600 China; 3 Dalian Customs Technology Center, Dalian, 116001 China

**Keywords:** *Vibrio vulnificus*, Aquatic products, Aquaculture waters, Visual, Loop-mediated isothermal amplification (LAMP)

## Abstract

**Background:**

A visual, rapid, simple method was developed based on a loop-mediated isothermal amplification (LAMP) assay to detect *Vibrio vulnificus* in aquatic products and aquaculture waters.

**Results:**

Genomic DNA was extracted from *Vibrio vulnificus* using the boiling method, and optimized primers were used to detect the *gyrB* gene using a visual LAMP method. The sensitivity of the assay was 10 fg/μL, and the obtained results were stable and reliable. Out of 655 aquatic product samples and 558 aquaculture water samples, the positive rates of *Vibrio vulnificus* detection were 9.01% and 8.60%, respectively, which are markedly higher than those of the traditional culture identification methods.

**Conclusion:**

The relatively simple technical requirements, low equipment cost, and rapid detection make the visual LAMP method for the detection of *Vibrio vulnificus* a convenient choice for field detection in the aquaculture industry.

## Background

*Vibrio* species are the most dominant bacteria in the marine environment and are widely distributed in estuaries, bays and coastal waters, as well as the body surface and intestinal tract of marine organisms [1]. Human infections with *Vibrio spp.* caused by the consumption of fish, shellfish, shrimp, crab and other aquatic products have become a worldwide concern [2, 3]. At least 12 pathogenic *Vibrio* species have been reported, which are not only a public health issue but also cause huge economic losses to the aquaculture industry [1–4]. *V. vulnificus* is responsible for more than 50% of infectious diseases in aquaculture [5, 6] and has the highest fatality rate of any foodborne pathogen [7–9].

*V. vulnificus* infection can be caused by eating raw or uncooked oysters [10]. *V. vulnificus* infections are characterized by acute onset, severe disease and high mortality, with 50% of patients dying as a result of multiple organ failure within 48 h after onset [11], increasing to 100% if patients are not treated within 72 h [12]. *V. vulnificus* infections tend to increase with increasing climate warming and offshore activities.

*V.vulnificus* is a thermophilic bacterium. When the water temperature is higher than 18 °C, *V.vulnificus* will rapidly multiply, reach the peak value at 26 °C, and enter the dormant state when the temperature is lower than 5 °C. Therefore, the peak season of *V.vulnificus* infection is in summer and autumn [13].

Different sampling types and sampling procedures also lead to different detection rates. In 2016, the detection rate of *V.vulnificus* in different types of aquatic products in Beijing was quite different, among which the detection rate of shrimp was as high as 52.38%, followed by 37.88% for shellfish and 22.22% for fish [4]. In 2015, the pollution rate of *V.vulnificus* in the samples of tegillarca granosa in Zhoushan city, China, was the highest in summer and autumn, while the pollution rate in the retail market was high in spring and winter, and the pollution level in winter was higher than that in livestock farms [14].

The detection of *V. vulnificus* in aquatic products is a challenge because it is difficult to isolate and grow under laboratory conditions and is readily inhibited by other *Vibrio* species. The technology used for biochemical identification of *Vibrio* is complex. The techniques are time-consuming and often require professional technicians. Bonny SQ et al. [15–18] established PCR and real-time fluorescence quantitative PCR methods for the detection of marine *vibrio* based on the 16S rRNA gene and VvhA gene of *Vibrio*. The PCR method has the advantage of strong specificity, but it requires specific experimental conditions, expensive equipment, and relatively complex operation; thus, it is not easy to popularize in aquaculture farms.

The GyrB gene, commonly found in bacteria, is a single-copy gene encoding DNA helicase B subunit protein, which plays an important role in the process of DNA replication. The gyrB gene is a suitable phylogenetic marker that can be used to study phylogenetic and taxonomic relationships at the species level of *vibrio* [19]. Venkateswaran [19] used gyrB sequence data to analyse the phylogenetic position of new *vibrio* isolates, and each group of new v*ibrio* isolates met the threshold standard of sequence diversity, providing a new basis for sequence diversity among different *vibrio* species.

Loop-mediated isothermal amplification (LAMP) is a simple and rapid technique for gene amplification that was developed by Notomi et al. (2000) [20]. It has the advantages of high specificity, efficiency and simple technical requirements. Compared with traditional PCR, LAMP can obtain a sufficient amount of target DNA for analysis within 1 h [21], and as a large amount of white magnesium pyrophosphate precipitate is produced in the LAMP reaction; the results can be determined by naked eye observation or turbidity meter, which is suitable for rapid detection in the field laboratory. LAMP has been widely used for the detection of pathogens [22–24]. Yamazaki [25, 26] and Chen [27] established a LAMP detection method for *V.parahaemolyticus* based on tlh, tdh, trh and toxR genes, respectively. In addition, multiple LAMP [28], in situ LAMP [29], real-time LAMP with multiple endonuclease restriction [30], LAMP-LFD (lateral flow dipstick) [31] and microfluidic LAMP [32] were also established by some researchers for *V.parahemolyticus*. Most of these studies focused on LAMP detection of *V.arahaemolyticus*. Ren [33] established a LAMP detection method for *V. vulnificus* based on cytolytic genes for the first time. Beichuangnan et al. [34] established a LAMP method for the detection of *V. vulnification* using haemolysin gene A (HA) and repeats in toxin (RTX) genes as targets. Currently,, there is a lack of visual LAMP detection methods for *V.vulnificus* that are suitable for promotion in aquaculture. In this study, we developed a visual LAMP-based method for the detection of *V. vulnificus* in aquatic products and environmental water samples with high specificity, sensitivity and reproducibility by targeting the *gyrB* gene.

## Results

### Optimized method for extraction of *Vibrio* genomic DNA

**Fig. 1:**
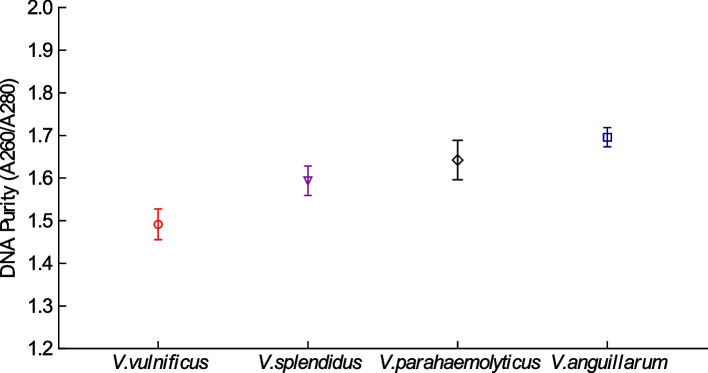
Genomic DNA purity indices (A_260_/A_280_) obtained from the samples. The purity indices (A260/A280) of the four *Vibrio* species from left to right can reach 1.5 and above

The genomic DNA of *V. vulnificus*, *V. splendidus*, *V. parahaemolyticus*, and *V. angularis* was extracted using boiling methods. The purity and concentration of each sample were evaluated using an ultramicro spectrophotometer (Table 1). As shown in Fig. 1, nucleic acid purity index values (A_260_/A_280_) of ≥ 1.5 or above were achieved by the boiling method.

**Table 1:** Absorption and concentration of genomic DNA extracted from four *Vibrio* species

*Vibrio* species	Mean ± standard deviation	
	A_260_/A_280_	Concentration(μg/mL)
*V. vulnificus*	1.594 ± 0.034	679.950 ± 16.193
*V. splendidus*	1.491 ± 0.036	356.800 ± 7.990
*V. parahemolyticus*	1.642 ± 0.046	399.500 ± 10.607
*V. anguillarum*	1.696 ± 0.023	422.050 ± 3.182

According to a previous report [35], if the magnesium ion is added to calcein before the LAMP reaction, the green fluorescence of calcein will be quenched, and the dye will become orange. After LAMP amplification, the pyrophosphate and manganese ions generated by the reaction combine and deposit, and the magnesium ion will have the opportunity to combine with calcein and affect the fluorescence signal of calcein. In such a case, the colour of a positive detector tube is observed as green fluorescence instead of the initial orange–red colour, and a negative detector tube will remain orange–red. Altogether, the final result will be solid green fluorescence in a positive reaction and weak green fluorescence in an adverse reaction when stimulated by 365 nm blue light.After staining the nucleus with fluorescent dye, quantitative measurement of the fluorescence intensity emitted by the cell can determine the content of DNA and RNA in the nucleus and analyse the cell cycle and cell proliferation. When using syto-9, a fluorescent chimeric dye with little influence on amplification, for real-time LAMP amplification, SYTO9 has a very low inhibition effect on PCR [36], and rearrangement does not occur during DNA unchain. This allows the fusion curves with these dyes to have a higher resolution so that the sources of multiple LAMP amplification products can be easily identified by fusion or annealing curve analysis.

**Fig. 2:**
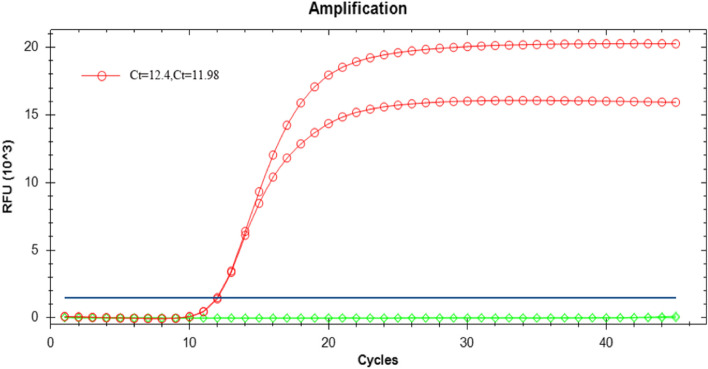
LAMP fluorescence amplification curve of genomic DNA isolated from *V. vulnificus* by boiling method. The CT values of parallel samples were 12.4 and 11.98 respectively

**Fig. 3:**
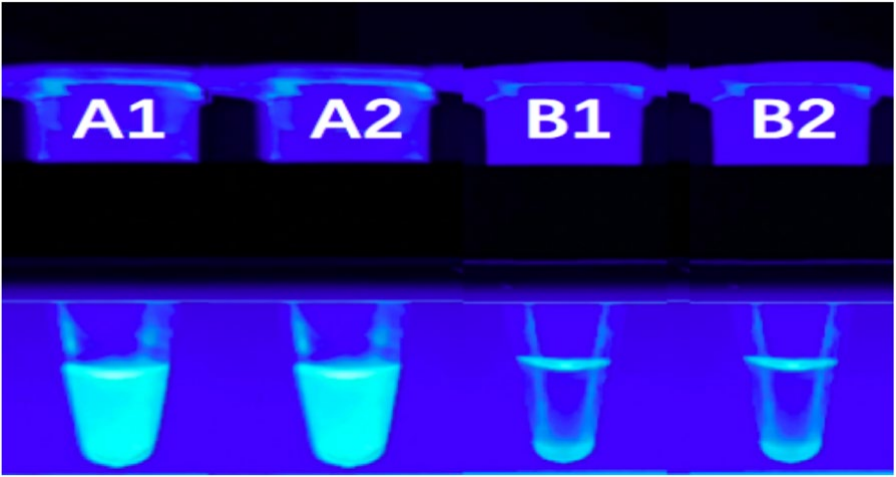
Visual detection of LAMP amplification products in genomic DNA isolated from *V. vulnificus*.The figure indicate fluorescence for genomic DNA of *V. vulnificus*. (A1) *V. vulnificus*. (A2) *V. vulnificus*.(B1) Blank control.(B2) Blank control

LAMP fluorescence amplification curves were generated for the extracted DNA after the addition of the SYTO-9 fluorescent dye (Fig. 2), and changes in the fluorescence intensity of the product were observed under UV light after the addition of MnCl_2_-calcein (Fig. 3). Typical LAMP fluorescence amplification curves were generated using *V. vulnificus* DNA extracted by the boiling method (Fig. 2), without difference between the Ct values obtained for each group. Typical changes in the fluorescence intensity were also observed using the visual dye method (Fig. 3). These findings indicate that the residual carbohydrates produced in the sample extracted using the boiling DNA cleavage method do not affect the LAMP reaction. Furthermore, this method has the advantages of rapid extraction, low cost and convenience. Therefore, we selected the boiling method for the extraction of *Vibrio* genomic DNA in this study.

### LAMP assay specificity

**Fig. 4:**
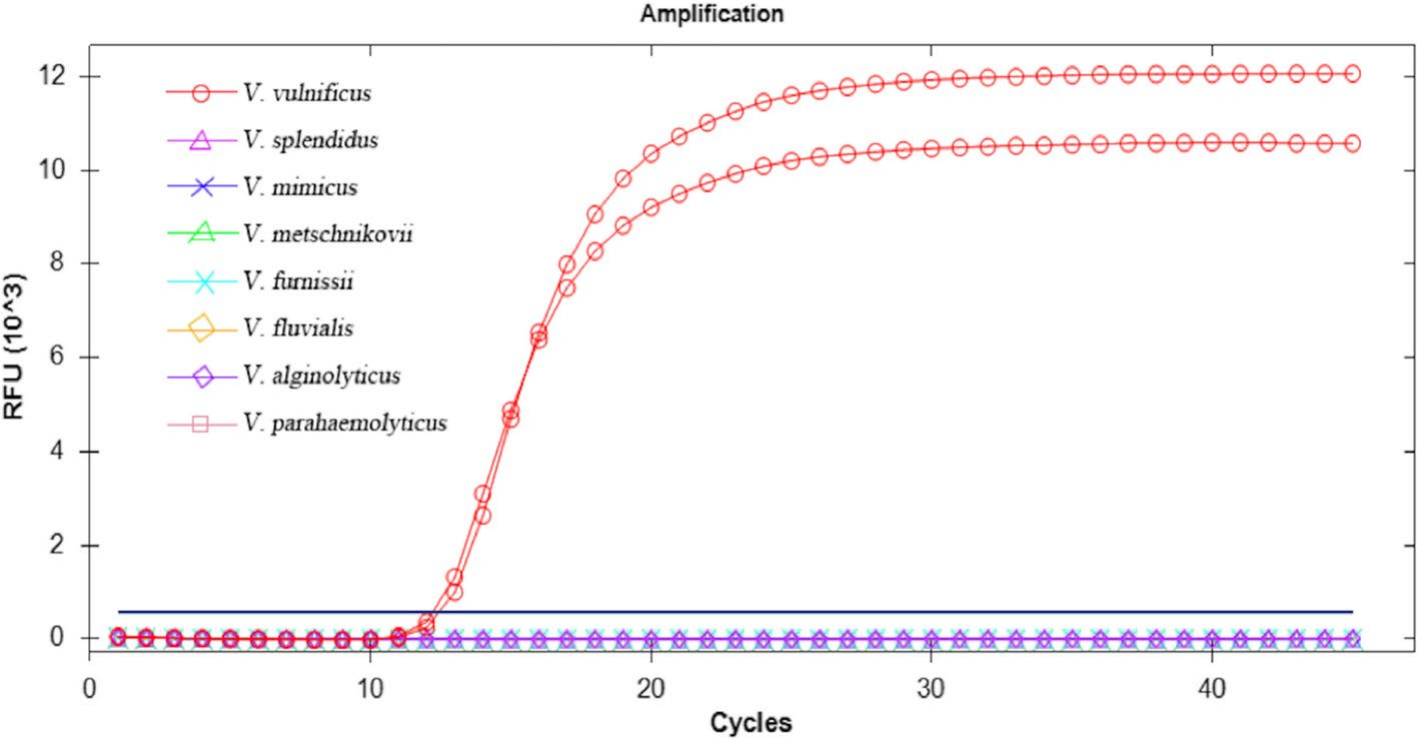
Specificity test for the detection of *V. vulnificus* by the LAMP fluorescence assay. The results indicate that *V.vulnificus* was a specific amplification curve, and other seven *Vibrio* species ware negative

**Fig. 5:**
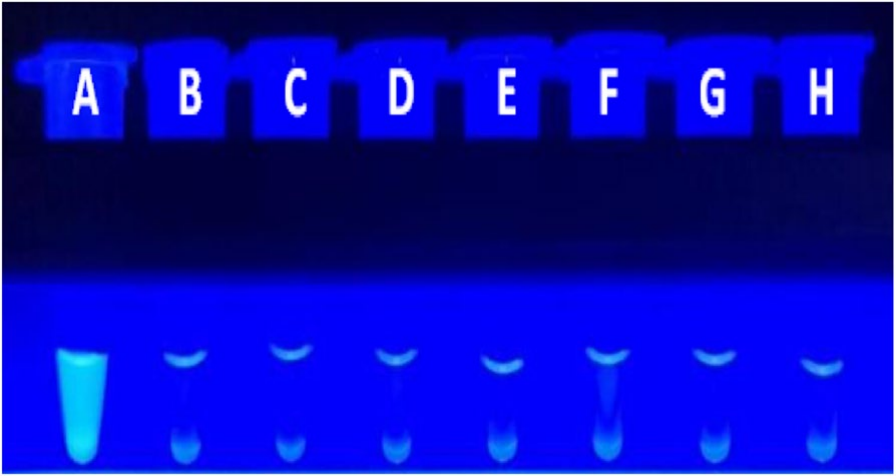
Specificity of the visual LAMP assay for the detection of *V. vulnificus* under UV light. The results indicate strong fluorescence intensity for *V.vulnificus*, and no fluorescence for other seven *Vibrio* species. **A**: *V. vulnificus*; **B**: *V. splendidus*; **C**: *V. mimicus*; **D**: *V. metschnikovii*; **E**: *V.furnissii*;**F**: *V. fluvialis*; **G**: *V. alginolyticus*; **H**: *V. parahaemolyticus*

The specificity of the selected primers was then evaluated for the detection of eight *Vibrio* species (*V. vulnificus*, *V. splendidus, V. mimicus*, *V. metschnikovii*, *V. furnissii*, *V. fluvialis*, *V. alginolyticus*, and *V. parahemolyticus*) using the fluorescence amplification curve (SYTO-9 fluorescent dye) and colour change (MnCl_2_-calcein) methods of LAMP amplification, as shown in Figs. 4 and 5, respectively. *V. vulnificus* was amplified specifically, while no amplification of the other *Vibrio* species was detected. Furthermore, the results obtained using the two detection methods were consistent. These findings indicate that these primers allow specific detection of *V. vulnificus* using the LAMP method.

### LAMP assay sensitivity

**Fig. 6:**
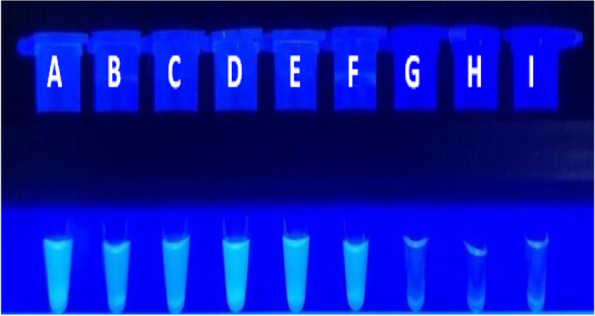
Sensitivity of the LAMP fluorescence method for the detection of *V. vulnificus*. The curves from left to right indicate decreasing concentrations of *V. vulnificus* genomic DNA [100 pg/ μL to 10 fg/μL per reaction]. **A**: 100 pg /μL; **B**: 10 pg/μL; **C**: 1 pg/μL; **D**: 100 fg/μL; E: 10 fg/μL

**Fig. 7:**
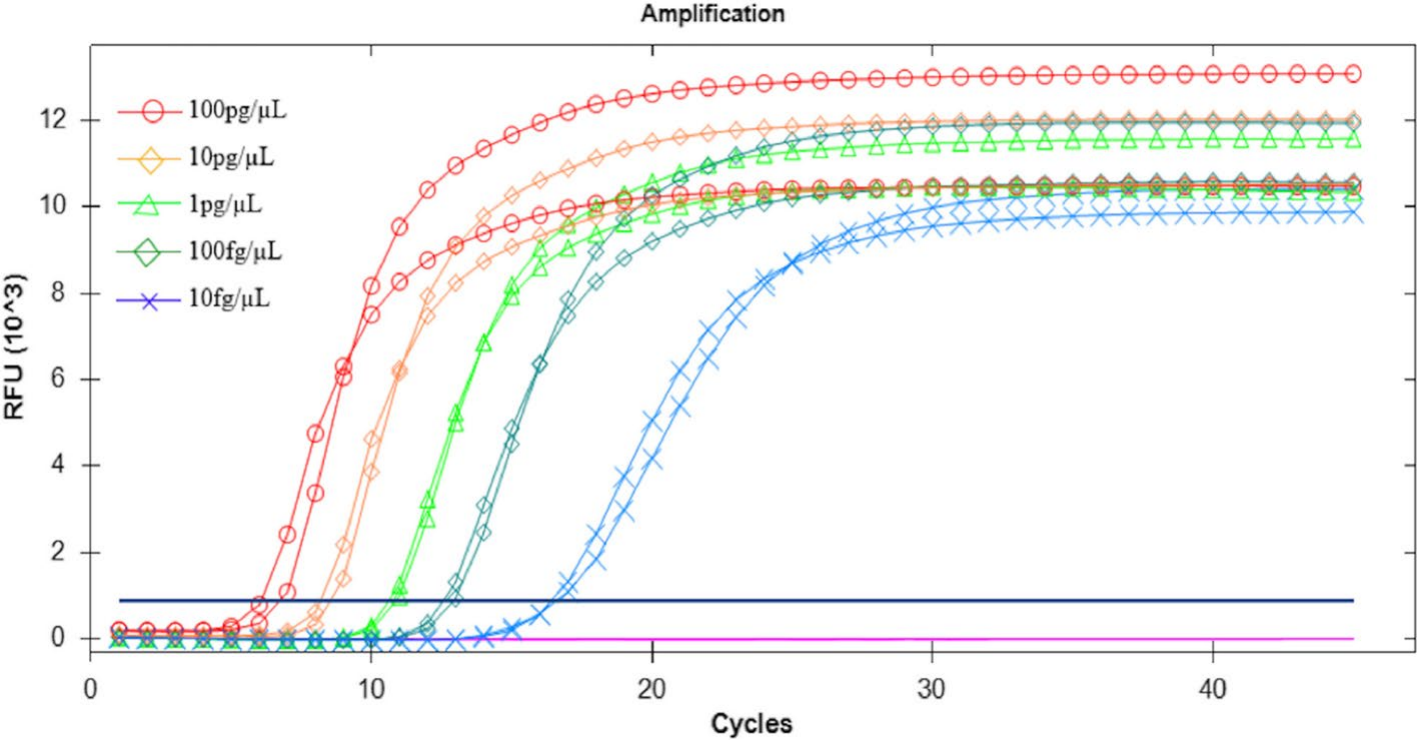
Sensitivity test for detection of *V.vulnificus* by visual LAMP method*.* The curves from left to right indicate decreasing concentrations of *V.vulnificus* genomic DNA from [1 ng/ μL to 0.1 fg/μL per reaction]. **A**: 1 ng/μL; **B**: 100 pg/μL; **C**: 10 pg/μL; **D**: 1 pg/μL; **E**: 100 fg/μL; **F**: 10 fg/μL; **G**: 1 fg/μL; **H**: 0.1 fg/μL; **I**: blank control

We compared this method with the PCR method to determine the detection sensitivity of this method. The obtained results are summarized in Table 2. The sensitivity of the LAMP assay for the detection of *V. vulnificus* using the optimized primers was evaluated using serial dilutions of the bacterial genomic DNA as templates. Using the LAMP reaction fluorescence amplification curve (with SYTO-9) method, the fluorescence amplification curves were consistent at concentrations of *V. vulnificus* genomic DNA ≥ 10 fg/μL, while the amplification was inconsistent and unstable at concentrations of ≤ 1 fg/μL (Fig. 6). Using the colour change (MnCl_2_-calcein) method, *V. vulnificus* amplification products were detected at concentrations of genomic DNA ≥ 10 fg/μL but not at concentrations of ≤ 1 fg/μL (Fig. 7). Table 2 shows that both the method established in this study and the PCR method achieve the same detection sensitivity. Thus, both LAMP methods can be used to detect *V. vulnificus* with a sensitivity of 10 fg/μL. The sensitivity results were basically consistent with those of the PCR method.

**Table 2:** Sensitivity results of the three methods

Sample name	Concentration	Results of fluorescence LAMP	Results of visual LAMP	Results of PCR
the bacterial genomic DNA	1 ng/μL	+	+	+
	100 pg/μL	+	+	+
	10 pg/μL	+	+	+
	1 pg/μL	+	+	+
	100 fg/μL	+	+	+
	10 fg/μL	+	+	+
	1 fg/μL	±	±	±
	0.1 fg/μL	ND	ND	ND
Minimum detected Concentration level		10 fg/μL	10 fg/μL	10 fg/μL

+ : positive; ± : weakly positive (one or two positive of three samples); ND: undetected

### Analysis of actual samples

The LAMP assay established in this study was then evaluated for the analysis of aquatic product samples and water samples. Among 655 samples of aquatic products, 59 samples (9.01%) were positive for *V. vulnificus* (Table 3)*.* Among 558 environmental water samples, 48 samples (8.60%) were positive for *V. vulnificus* (Table 4). Furthermore, consistent results for the detection of *V. vulnificus* in aquatic product and environmental water samples were obtained using the fluorescence amplification curve (with SYTO-9 fluorescent dye) and colour change (with MnCl_2_-calcein) methods.

**Table 3:** LAMP detection of *V. vulnificus* in different aquatic products

Sample type	Sampling quantity (pieces)	Number of positive results of *V. Vulnificus*, n/number of total samples, %		
		Results of the fluorescence amplification curve	Results of colour change	Positive rate (%)
Freshwater shrimp and crab	36	0	0	0
Freshwater fish	105	6	6	5.71
Seawater shrimp and crab	53	6	6	11.32
Seawater fish	171	12	12	7.02
Shellfish	189	35	35	18.52
Cephalopods	101	0	0	0
Total	655	59	59	9.01

**Table 4:** LAMP detection of *V. vulnificus* in environmental water samples

Sample	Sampling quantity (pieces)	Number of positive results of *V. Vulnificus*, n/number of total samples, %		
		the results of the fluorescence amplification curve	the results of colour change	Positive rate (%)
Sea water	440	45	45	10.23
River water	98	2	2	2.04
Aquaculture water	20	1	1	5.00
Total	558	48	48	8.60

Validation of the LAMP results by real-time fluorescent PCR [4] revealed 100% consistency between the two methods. Furthermore, *V. vulnificus* samples cultured in vitro were detected with 83.76% positivity (*P* = 0.00002). The results of this study showed that the rate of *V. vulnificus* detection in aquatic products and environmental water samples using biochemical methods was significantly lower than that achieved using the LAMP method. This discrepancy can be accounted for by the slow growth of many *Vibrio* isolates in vitro, which limits detection using biochemical methods.

### Detection of *V. vulnificus* in different types of samples

We also analysed the detection rates of *V. vulnificus* in 655 aquatic product samples comprised of pools of DNA obtained from different numbers of biological samples using the LAMP assay (Table 3). The highest positive detection rate was obtained for the pool of 35 shellfish samples (18.52%; 35/189), indicating that *V. vulnificus* is enriched in shellfish. Furthermore, the positive detection rate of *V. vulnificus* in shellfish samples was significantly higher than that in seawater fish samples (*χ*^2^ = 10.461, *P* < 0.01), freshwater fish samples (*χ*^2^ = 9.221, *P* < 0.01) and freshwater shrimp and crab samples (*χ*^2^ = 7.895, *P* < 0.01). There was no significant difference in the positive detection rates of cephalopod samples (*χ*^2^ = 21.271, *P* < 0.01) or sea shrimp and crab samples (*χ*^2^ = 1.524, *P* > 0.05).

Similar analysis of the 558 environmental water samples (Table 4) showed that the positive *V. vulnificus* detection rates for seawater, river water and aquaculture water were 10.23%, 2.04% and 5.00%, respectively. Furthermore, the positive rate of *V. vulnificus* detection in seawater samples was significantly higher than that in river water samples (*χ*^2^ = 6.737, *P* < 0.01), whereas there was no significant difference in the positive rate between the aquaculture and river water samples (*P* > 0.05).

### Detection of *V. vulnificus* in samples collected at different times of the year

Previous studies have shown that the positive detection rate of *V. vulnificus*, which is a thermophilic bacterium, increases as the water temperature rises throughout the year, with the highest detection rate in summer [37]. In our analysis of samples collected at different times of the year, the highest positive *V. vulnificus* detection rate (29.79%) was observed between June and August, which was 29.79% (Table 5).

**Table 5:** LAMP detection of *V. vulnificus* in samples collected at different times of the year

Sampling time	Sampling quantity (pieces)	Number of positive results of *V. Vulnificus*, n/number of total samples, %		
		Results of the fluorescence amplification curve	Results of colour change	Positive rate (%)
March–May	174	18	18	10.34
June–August	235	70	70	29.79
September– November	132	17	17	28.03
December– February	114	2	2	1.75
Total	655	107	107	16.34

### Detection of *V. vulnificus* in samples obtained at different stages of the sales process

Most farmers’ markets in China operate based on open management and sales models. Compared with farmers’ markets, the conditions in supermarkets will be more standardized, with better sanitation and less cross-contamination between goods. In accordance with this, we found that the average rate of *V. vulnificus* contamination of samples from farmers’ markets was higher (30.01%; 68/206) than that in supermarkets (7.41%; 14/189) (Table 6).

**Table 6:** LAMP detection of *V. vulnificus* in samples from different sampling links

Sampling link	Sampling quantity (pieces)	Number of positive results of *V. Vulnificus*, n/number of total samples, %		
		the results of the fluorescence amplification curve	the results of colour change	Positive rate (%)
Café	155	18	18	11.61
Supermarket	189	14	14	7.41
Farmers’ market	206	68	68	33.01
Online store	105	7	7	6.67
Total	655	107	107	16.34

## Discussion

*V.vulnificus* is widely distributed in marine environments and seafood; infection typically occurs through ingestion or through wounds; clinical symptoms mainly include gastroenteritis and festering wound necrotic lesions, which easily cause sepsis [38–40]. Therefore, *V.vulnificus* has become the world's dominant ocean pathogenic bacterium, and deputy haemolytic *Vibrio* and human pathogenic *V.cholerae* are listed as the major *Vibrio* species. With the increasing demand for seafood and the pursuit of sports at sea, there are an increasing number of cases of contact and infection with *V.vulnificus.* From 1996 to 2010, more than 1600 cases were reported in the United States, with a fatality rate of 30% [41]. From 2003 to 2010, nearly 100 cases were reported in Taiwan, with a fatality rate of 60% [42].

Currently, the methods used to detect *V. vulnificus* include identification of culture medium and morphology. Although these traditional methods do not require expensive instruments, they are time-consuming and have poor specificity and sensitivity [42]. Although fluorescence quantitative PCR detection, conventional PCR detection and ELISA detection have strong specificity and sensitivity, they need to be operated by professionals and require expensive equipment; thus, these approaches are not conducive to the promotion of grassroots detection. To ensure the safety of edible seafood, it is necessary to establish a fast, simple and practical method to meet the detection requirements.

LAMP is a constant temperature nucleic acid amplification technology that not only has good specificity and sensitivity [43] but also does not require very expensive equipment or professional technicians to operate and allows directly observe the results with the eyes. The greatest advantage of LAMP is the short detection time, which can greatly reduce the detection time and cost [44]. In this study, the conserved sequence of the gyrB gene of the *V.vulnificus* gene was used as the target sequence, and inner, outer and ring primers were designed to establish a rapid detection method for *V.vulnificus*. The detection results were compared with the PCR detection results, and the results showed that the sensitivity of the method was higher than that of ordinary PCR detection, and the time was shorter than that of PCR. The specificity detection results showed that the established LAMP had high specificity, and the detection results could be achieved intuitively by observing precipitation with the eyes. Objective bacterial DNA can be obtained by the boiling method, avoiding the purchasing of a DNA extraction kit and further reducing the cost of detection.

The LAMP assay established in this study has the advantages of simplicity, speed, high specificity, good sensitivity, simple judgement results and low cost, which is conducive to the promotion and application of LAMP at the basic level. Through further study and exploration, LAMP can become a routine method to detect the safety of seafood.

## Conclusions

In this study, we established a LAMP-based method for the rapid (within 30 min) detection of *V. vulnificus* in aquatic products (9.01%) and environmental water (8.60%) in different seasons and from different commercial sources, such as farmers’ markets and supermarkets. This technique provides an important resource to ensure the safety of edible aquatic products and environmental water.

In a study of 105 samples of seafood randomly collected in Beijing markets, Wang et al. [4] reported the accuracy of *V. vulnificus* detection in 100% and 67.50% of samples by real-time fluorescent PCR and VITEK methods, respectively. In this study, we established a visual LAMP-based method for the detection of *V. vulnificus* and confirmed the applicability of this approach for aquaculture field monitoring by analysing 655 aquatic product samples and 558 environmental water samples. We found that the coincidence rate of results obtained using the visual LAMP-based and real-time PCR methods was 100%, while the coincidence rate between this method and classical biochemical culture identification was 83.76% (*P* = 0.00002). Furthermore, the positive *V. vulnificus* detection rate of the visual LAMP-based detection method was significantly higher than that of the classical isolation and culture identification method.

In particular, the visual LAMP-based detection method developed in this study provides a simple, rapid and economical technique that can be applied to the detection of V. vulnificus in the field and will be important in the prevention and control of V. vulnificus infections in aquaculture.

## Methods

### *Vibrio* species

The following *Vibrio* species were used in this study: *V. vulnificus* ATCC 27,562, *V. splendidus* ATCC 33,125, *V. mimicus* CICC 21,613, *V. metschnikovii* ATCC 27,562, *V. furnissii* IQCC 12,309, *V. fluvialis* CICC 21,612, *V. alginolyticus* ATCC17749, *V. parahaemolyticus* ATCC 17,802, and *V. anguillarum* CICC 10,475. These 9 *vibrio* species are common and pathogenic marine *vibrio* species. All *Vibrio* species were stored by the Microbiology Laboratory, Dalian Customs Technology Center (Dalian, China) and were identified using biochemical methods and stored at -80 ± 1 °C.

### Sample preparation

Environmental water samples (500 mL) were collected from rivers (upper, central, and lower parts) and the sea. For each sample, 1 mL of water was added to a tube containing 9 mL of alkaline peptone broth (APB) with 3% NaCl. For marine shellfish, the shells were washed with running water and sterilized with 70% alcohol, and approximately 20 g was homogenized in 50 mL of 0.85% sterile normal saline. Infected fresh water or marine fish were sterilized with 70% alcohol before the liver, spleen, kidney and ulcerative lesions were removed. Approximately 20 g of each tissue was homogenized. Shrimp and crab were sterilized with 70% alcohol before samples (20 g) were homogenized, and 1 mL of the homogenate was added to 9 mL of APB with 3% NaCl. Then, the samples were incubated overnight at 37 ± 1 °C to amplify the bacteria. Subsequently, 1 mL of the culture was centrifuged at1204g for 2 min, and the supernatant was collected for DNA extraction. The culture mixture was used to inoculate TCBS agar plates using a sterilized loop and incubated overnight at 37 ± 1 ℃ for the identification of *Vibrio*.

### DNA extraction

Bacterial genomic DNA was extracted using the boiling method [33]. For the boiling method, the samples (10 mg) were mixed with 100 μL of lysis buffer, vortexed and heated at 95 °C for 10 min before centrifugation at 1204 g for 5 min as previously described. The supernatant containing the genomic DNA was transferred to a new microtube and stored at -20 °C for downstream applications.

### Primer design and synthesis

Homology analysis of the *gyrB* gene (GenBank ID: MN540397.1) was performed by DNAStar software. Six LAMP primers were designed based on the *gyrB* gene using Primer Explorer V4 software (Eiken Chemical Co., Ltd., Japan) (Table 7). These primers were synthesized by TaKaRa (Dalian, China).

**Table 7:** Sequence of LAMP primers for the *gyrB* gene of *V.vulnificus* in this work

Primer	Sequence (5′-3′)	Position	Length
F3	GCTTGCTATCATCGGTGAT	72–90	19
B3	CACCGTCACGCTGTG	451–437	15
FIP	AACGCTTGAATACCACCTTCATACAATCCTAGCGAAGCGTCT	287–263	25
BIP	TCACTCACTTGAACCGCAACAGCCACTTCAACCGCAA	290–310	21
LOOP F	CGACACGCCAGAGTTCA	216–200	17
LOOP B	TTAATGCCGAGCGTGAAGA	341–359	19

### LAMP reaction system and conditions

Fluorescence LAMP assays were performed in a 25-μL reaction volume containing 12.5 μL of 2 × RM reaction solution, 1.0 μL of *Bst* DNA polymerase, 0.5 μL of SYTO-9 fluorescent dye (Life Technologies), 1.0 μL of each primer (final concentrations: 0.4–1.6 μM for inner primers and 0.1–0.2 μM for outer primers, and 0.1–0.8 μM for loop primers), 2.0 μL of DNA template and 6.0 μL of ddH_2_O.

Visual LAMP assays were performed in a 25-μL reaction volume containing 12.5 μL of 2 × RM reaction solution, 1.0 μL of *Bst* DNA polymerase, 1.0 μL of visual MnCl_2_-calcein stock solution (Merck), 1.0 μL of each primer (final concentrations: 0.4–1.6 μM for inner primers and 0.1–0.2 μM for outer primers, and 0.1–0.8 μM for loop primers), 2.0 μL of DNA template and 5.5 μL of ddH_2_O.

The CFX96 Touch Real-Time PCR Detection System (Bio-Rad) was used to observe the fluorescence amplification curve (with SYTO-9 fluorescent dye) using the following reaction conditions: 63 °C for 15 s, followed by 45 cycles at 63 °C for 45 s.

For the visual LAMP assay, MnCl_2_-calcein was added to the reaction mixture using the following reaction conditions: 65 ℃ for 30 min, followed by 95 ℃ for 2 min or ice for 2 min. The obtained result was observed under UV light (240–260 nm or 350–370 nm). Samples that turned green were considered positive for *V. vulnificus*, while samples that remained orange were considered negative.

### Assay specificity and sensitivity

To verify the specificity, the LAMP assay was performed as described above using genomic DNA from *V. vulnificus*, *V. splendid*, *V. mimicis* [45], *V. metschnikovii, V. fischeri, V. fluvibrio, V. algolyticus* and *V. parahaemolyticus.* The sensitivity of the LAMP assay was determined by amplification of tenfold serial dilutions of *V. vulnificus* genomic DNA (1 ng/μL, 100 pg/μL, 10 pg/μL, 1 pg/μL, 100 fg/μL, 10 fg/μL, 1 fg/μL, and 0.1 fg/μL); the assay was repeated twice for each dilution. The sensitivity of the LAMP assay was compared with that of the PCR method.

### Application of detection of aquatic products and environmental waters

Samples of aquatic products (*n* = 655) obtained from restaurants (*n* = 155), supermarkets (*n* = 189), farmers’ markets (*n* = 206), and online stores (*n* = 105) and environmental water samples [*n* = 558; sea water (*n* = 440), river water (*n* = 98) and 20 aquaculture sea water (*n* = 20)] were also analysed using the visual LAMP detection method. Positive samples were isolated and cultured for biochemical identification of *V. vulnificus*.

### Statistical analysis

SPSS (Statistical Product and Service Solutions, IBM) software was used to perform chi-squared (*χ*^2^) tests, and Mann–Whitney tests were used to evaluate the significance of the difference between the results obtained using the two detection methods. *P* < 0.05 was considered to indicate statistical significance.

## Data Availability

All the data required are included in the manuscript.
